# ﻿Three new species of Entomobryidae (Collembola, Entomobryoidea) from China

**DOI:** 10.3897/zookeys.1167.104090

**Published:** 2023-06-22

**Authors:** Mei-Dong Jing, Yi-Tong Ma

**Affiliations:** 1 School of life Sciences, Nantong University, Jiangsu 226000, China Nantong University Jiangsu China

**Keywords:** *Akabosia*, chaetotaxy, *Entomobrya*, *Homidia*, springtails, taxonomy

## Abstract

Three new species of entomobryid springtails (Collembola) from China are described here. *Homidiapseudozhangi***sp. nov.** is characterised by a narrow irregular longitudinal stripe on the body, smooth chaetae e and l_1_ of the labial base, and the relative position of the **s**pecialized microchaeta on Abd. I; *H.qianensis***sp. nov.** by its colour pattern on the antennae and nine sutural macrochaetae on the head; and *Entomobryashaanxiensis***sp. nov.** by its colour pattern, labral papillae and the lateral process of labial papilla E. Specimens of *Akabosiamatsudoensis* Kinoshita, 1919 from China are redescribed, including description of some characters for the first time.

## ﻿Introduction

The genus *Homidia* Börner, 1906 is characterised by the presence of inner spines at the base of the dens, a transversal line of macrochaetae on the anterior part of Abd. IV, and a bidentate mucro with the subapical tooth much larger than the apical one. It is close to the genus *Sinhomidia* Zhang, 2009 (Zhang et al. 2009), but there are no scales in *Homidia*. Among 77 *Homidia* species of the world, 50 species were reported from China (Bellinger et al. 1996–2023).

As one of the largest genera in Collembola, *Entomobrya* Rondani, 1861 presents about 270 species, but only 19 species have been reported from China. It is characterised by scales absent, dorsal chaetotaxy polymacrochaetotic, mucronal subapical tooth subequal to the apical and dens without spines (Jordana 2012).

The genus *Akabosia* Kinoshita, 1919 is characterised by a crenulated dens and a rectangle mucro. It is close to the genus *Salina* MacGillivray, 1894, but the dens is not crenulated in the latter. These two genera belong to the subfamily Salininae of Entomobryidae (Godeiro et al. 2022). *Akabosia* contains only one species recorded from Japan (Kinoshita 1919; Yosii 1954, 1965), Korea (Mitra 1977) and China (Zhang et al. 2015).

## ﻿Materials and methods

Specimens were collected with an aspirator and stored in 99% alcohol. They were mounted on glass slides in Marc André II solution, and were studied with a Leica DM2500 phase contrast microscope. Photographs were taken under a Leica DFC300 FX digital camera mounted on the microscope and a ZEISS Gemini SEM 300, and enhanced with Photoshop CS2 (Adobe Inc.). The nomenclature of the dorsal macrochaetotaxy of the head are described follows Jordana and Baquero (2005) and the interocular chaetae follows Mari-Mutt (1986). Labial chaetae are designated following Gisin (1964) and tergal chaetae of the body following Szeptycki (1979) and Zhang et al. (2019).

### ﻿Abbreviations

**Ant.** antennal segment;

**Th.** thoracic segment;

**Abd.** abdominal segment;

**Mac** macrochaeta(e);

**Mes** mesochaeta(e);

**Ms** specialized microchaeta(e);

**Sens** specialized ordinary chaeta(e);

**NTU** Nantong University.

### ﻿Taxonomic account

**Figure 1.**
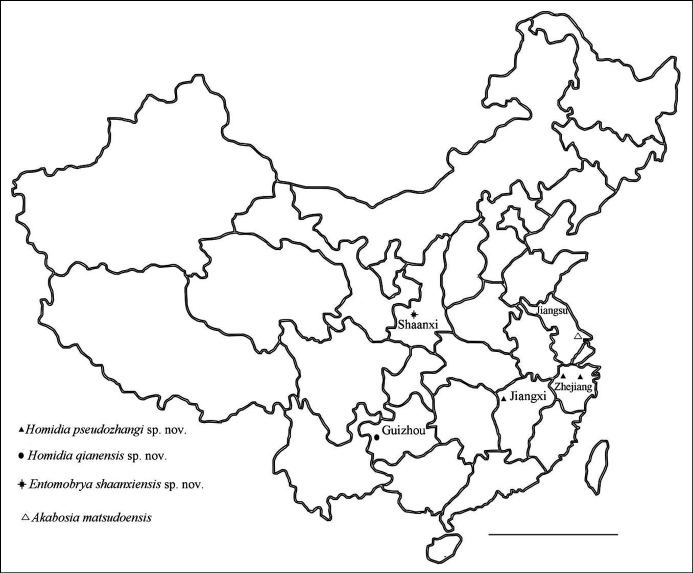
Distribution of the species described in the present paper. Scale bar: 1000 km.

Distribution in China of the species described present paper is shown in Fig. 1.

#### ﻿ Homidia pseudozhangi sp. nov.

Taxon classificationAnimaliaCollembolaEntomobryidae

992AC2A2-1D85-5C1D-AC20-123A1F225186

https://zoobank.org/DB6F4316-B891-4520-A592-10613EF0DF59

Figs 2–6 , 7–14 , 15–20 , 21 , 22, 23 , 24–30 , Table 1

##### Type material.

***Holotype*.** ♀ on slide, China, Zhejiang Province, Hangzhou City, Xihu District, Jiuxi Bus Station, 30°11'25″N, 120°06'47″E, 29 m asl, sample number 1183, collected by Y-T Ma, 6-VII-2018, from leaf litter, deposited in NTU. ***Paratypes*.** 4♀♀ on slides, same data as holotype.

##### Additional records.

3♀♀ on slides, China, Zhejiang Province, Hangzhou City, Linan District, Gate of Tianmu Mountain, 30°18'31″N, 119°26'44″E, 275 m asl, sample number 1181, collected by Y-T Ma, 14-VII-2018, from leaf litter; 3♀♀ on slides, CHINA, Jiangxi Province, Yichun City, Tonggu County, Tonggu Park, 28°31'54″N, 114°22'36″E, 239 m asl, sample number 1235, collected by Y-T Ma, 14-XI-2020, from leaf litter. All deposited in NTU.

##### Description.

Size. Body length up to 2.31 mm.

**Figures 2–6.**
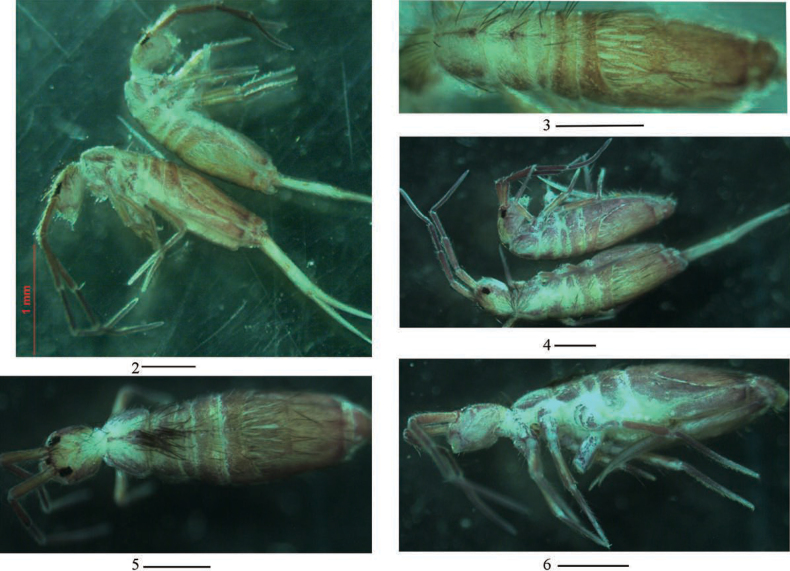
Habitus of *Homidiapseudozhangi* sp. nov. **2, 4** lateral view **3, 5** dorsal view **6** ventral view. Scale bars: 500 μm.

***Coloration*.** Ground colour pale white to pale yellow. Eye patches dark blue. Antennae gradually darker from Ant. I to Ant. IV. Brown to blue-violet pigment present on head anteriorly and laterally, thorax laterally and abdomen entirely, legs, ventral tube and basal manubrium. A narrow midline irregular longitudinal stripe present on midline from Th. II to Abd. I or II (Figs 2–6).

**Figures 7–14.**
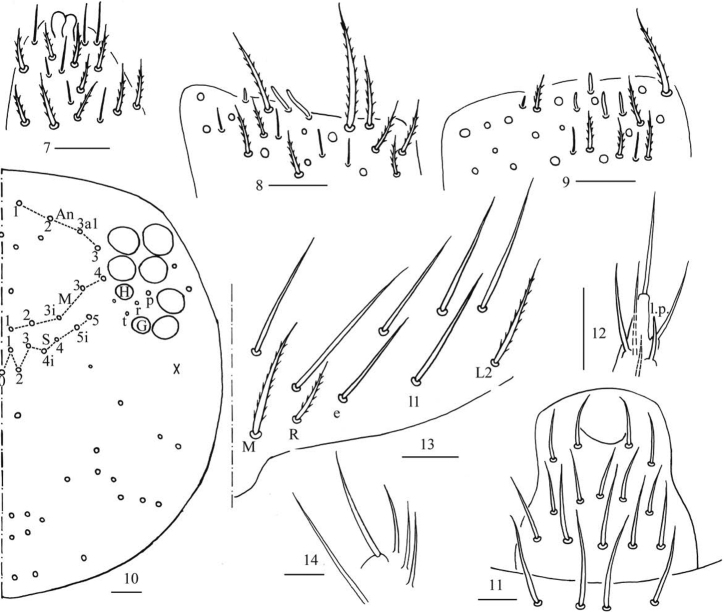
*Homidiapseudozhangi* sp. nov. **7** apex of Ant. IV **8**Ant. III organ **9** distal Ant. II **10** dorsal chaetotaxy of head **11** labrum **12** labial palp E **13** labial base **14** maxillary outer lobe. Scale bars: 20 μm.

***Head*.** Antenna 0.67–1.00 times body length; antennal segment ratio I: II: III: IV = 1: 1.23–1.50: 1.00–1.29: 1.43–2.43. Apical bulb of Ant. IV bilobed (Fig. 7). Ant. III organ with two distal rod-like chaetae ventrally (Fig. 8). Ant. II with four distal rod-like chaetae ventrally (Fig. 9). Eyes 8 + 8, G and H smaller than others; interocular chaetae with p, r, and t mes. Dorsal cephalic chaetotaxy with four antennal (An1, An2, An3a1, An3), five median (M1, M2, M3i, M3, M4) and eight sutural (S0, S1, S2, S3, S4, S4i, S5i, S5) mac (Fig. 10). Labral chaetae as 4/5, 5, 4, all smooth; labral papillae absent (Fig. 11). Lateral process (l.p.) of labial papilla E differentiated, as thick as normal chaeta, with tip not reaching apex of papilla E (Fig. 12). Chaetal formula of labial base as MRel_1_L_2_, chaetae e and l_1_ smooth, others ciliate, R/M as 0.40–0.67 (Fig. 13). Basal chaeta of maxillary outer lobe thin, subequal to apical one; sublobal plate with three smooth chaeta-like processes (Fig. 14).

**Figures 15–20.**
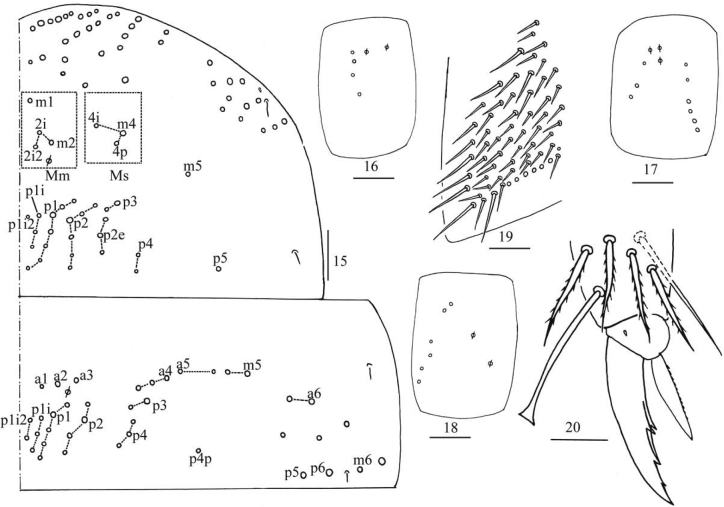
*Homidiapseudozhangi* sp. nov. **15** chaetotaxy of Th. II–III **16–18** coxal chaetotaxy of fore, middle and hind leg **19** trochanteral organ **20** hind foot complex. Scale bars: 50 μm (**15**); 20 μm (**16–20**).

***Thorax*.**Th. II with four medio-medial (m1, m2, m2i, m2i2), three medio-sublateral (m4, m4i, m4p), 23–32 posterior mac, one ms and two sens (ms antero-internal to sens). Th. III with 30–43 mac and two sens (Fig. 15). Pseudopores on coxa I–III as 2, 3, 2, respectively; coxal macrochaetal formula as 4 (rarely 3)/4+2, 3/4+2 (Figs 16–18). Trochanteral organ with 37–68 smooth spiny chaetae (Fig. 19). Tenent hair capitate and almost subequal to inner edge of unguis in length. Unguis with four inner teeth and distal one very faint, basal pair located at 0.40–0.49 distance from base of inner edge of unguis, distal unpaired teeth at 0.67–0.73 and 0.83–0.88 distance from base, respectively; unguiculus lanceolate, outer edge slightly serrate (Fig. 20).

**Figure 21.**
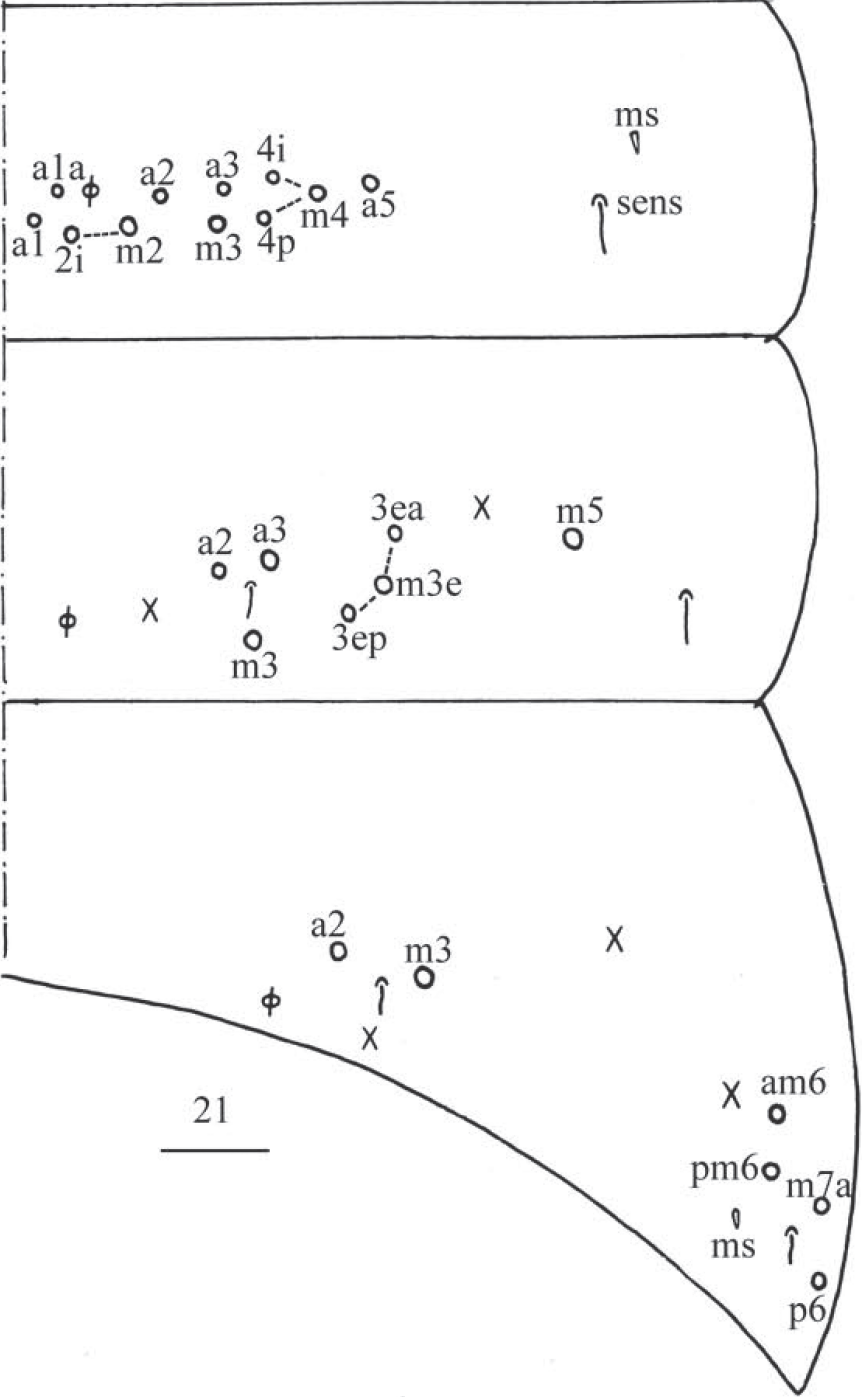
*Homidiapseudozhangi* sp. nov. Chaetotaxy of Abd. I–III. Scale bar: 50 μm.

**Figures 22, 23.**
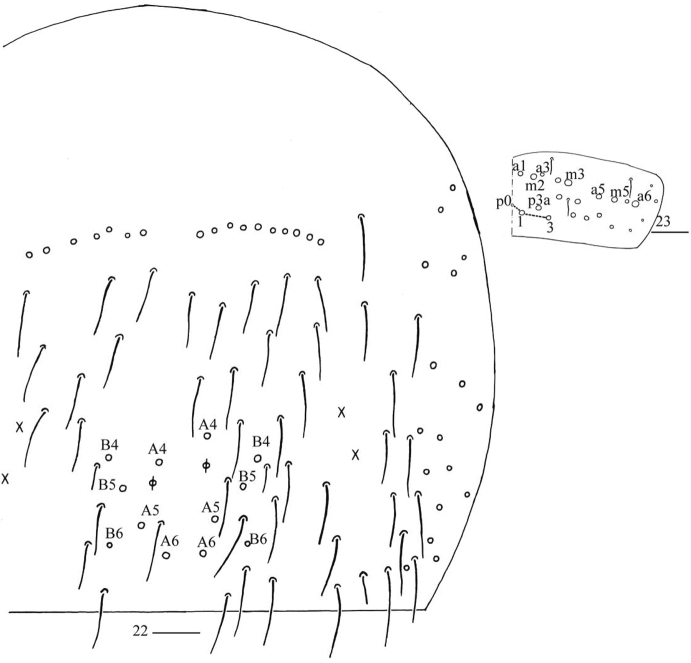
*Homidiapseudozhangi* sp. nov. **22** chaetotaxy of Abd. IV **23** chaetotaxy of Abd. V. Scale bars: 50 μm (**22**); 20 μm (**23**).

**Figures 24–30.**
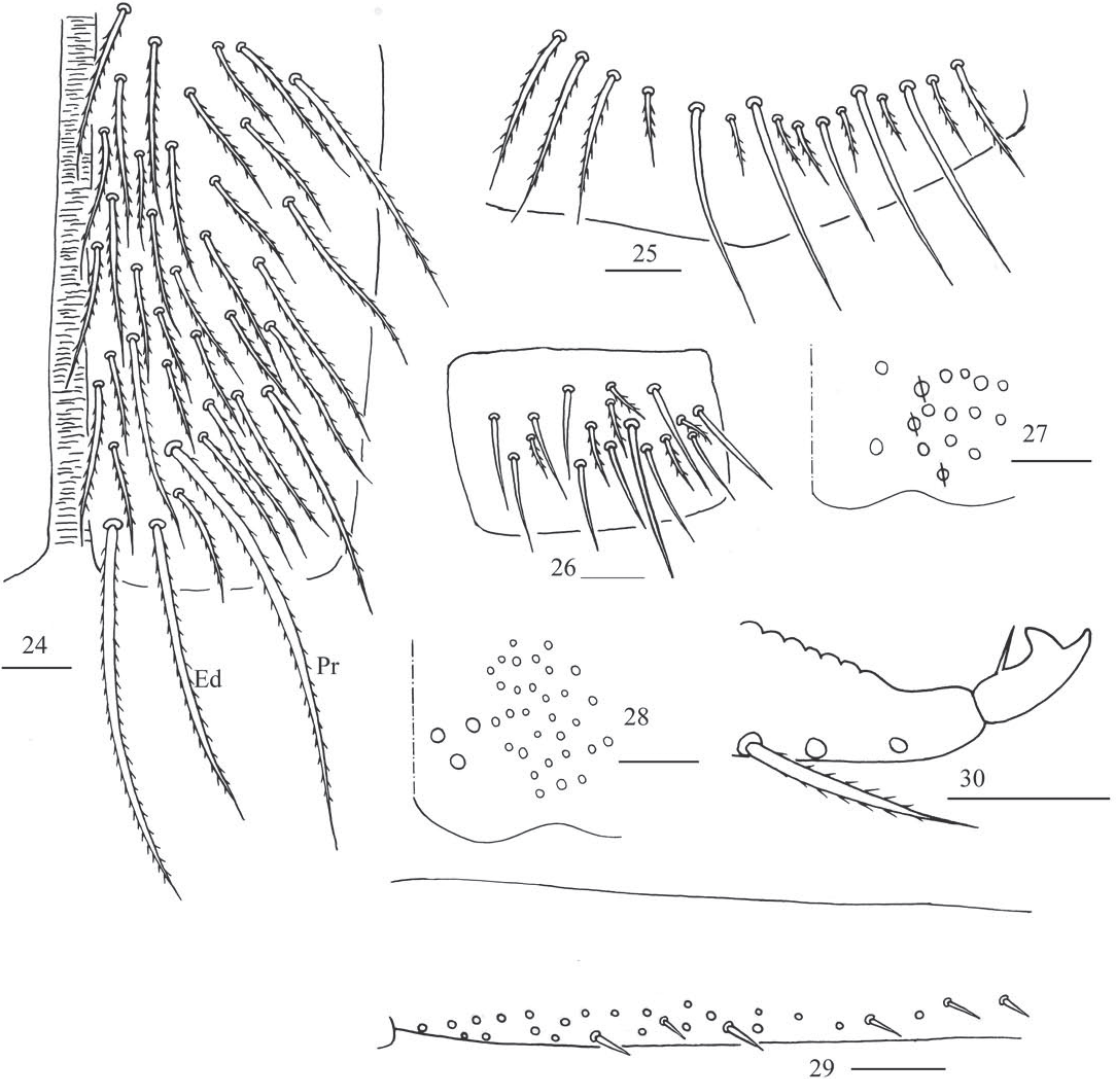
*Homidiapseudozhangi* sp. nov. **24** anterior face of ventral tube **25** posterior face of ventral tube apically **26** lateral flap of ventral tube **27** manubrial plaque **28** ventro-apical part of manubrium **29** proximal section of dens (circles also representing spines) **30** mucro. Scale bars: 20 μm.

***Abdomen*.** Range of Abd. IV length as 5.33–6.90 times as dorsal axial length of Abd. III. Abd. I usually with 11 (a1–3, a5, m2–4, m2i, m4i, m4p, a1a and a1 rarely absent), ms antero-external to sens. Abd. II with six (a2, a3, m3, m3e, m3ea, m3ep) central, one (m5) lateral mac and two sens. Abd. III with two (a2, m3) central and four (am6, pm6, m7a, p6) lateral mac, one ms and two sens (Fig. 21). Abd. IV with two normal sens and about half length of elongate sens; anteriorly with 7–12 mac arranged in irregular transverse row, posteriorly with six central mac (A4, A5, A6, B4, B5, B6), laterally with 17–24 mac (Fig. 22). Abd. V with three sens, middle one posterior to m3 (Fig. 23). Anterior face of ventral tube with 31–42 ciliate chaetae, 3+3 of them as mac, line connecting proximal (Pr) and external-distal (Ed) mac oblique to median furrow (Fig. 24); posterior face with numerous ciliate chaetae and 4–5 smooth chaetae apically (Fig. 25); lateral flap with 7–11 smooth and 6–10 ciliate chaetae (Fig. 26). Manubrial plate dorsally with 8–13 ciliate chaetae and three pseudopores (Fig. 27); ventrally with 21–34 ciliate chaetae on each side (Fig. 28). Dens with 28–39 smooth inner spines (Fig. 29). Mucro bidentate with subapical tooth larger than apical one; tip of basal spine reaching apex of subapical tooth; distal smooth section of dens almost equal to mucro in length (Fig. 30).

##### Etymology.

Named after its similar species *H.zhangi* (pseudo+zhangi).

##### Ecology.

In the leaf litter

##### Remarks.

The new species is characterised by a narrow irregular longitudinal stripe on the body, smooth chaetae e and l_1_ on the labial base and ms antero-external to the sens on Abd. I. It is similar to *H.acutus* Jing & Ma, 2022, *H.mediofascia* Shi, Pan & Bai, 2009, *H.yangdangensis* Pan, 2015 and *H.zhangi* Pan & Shi, 2012 in colour pattern, but can be separated from them by the smooth chaetae on the labial base, the relative position of ms on Abd. I and other characters. It is also similar to *H.phjongjangica* Szeptycki, 1973, *H.sichuanensis* Jia, Zhang, Zhao & Jordana, 2010 and *H.sinensis* Denis, 1929 in body chaetotaxy, but there are some differences between them in colour pattern, coxal macrochaetal formula and other characters (Table 1).

**Table 1.** Comparison between *H.pseudozhangi* sp. nov. and similar species.

Characters	*H.pseudozhangi* sp. nov.	*H.acutus* Jing & Ma, 2022	*H.mediofascia* Shi, Pan & Bai, 2009	*H.yangdangensis* Pan, 2015	*H.zhangi* Pan & Shi, 2012	*H.phjongjangica* Szeptycki, 1973	*H.sichuanensis* Jia, Zhang, Zhao & Jordana, 2010	*H.sinensis* Denis, 1929
Medial stripe on Th. II–III	narrow	absent	narrow	wide	absent	absent	absent	absent
Colour patches on Abd. II–IV	present	present	almost absent	present	present	present	present	present
Chaeta L_1_ on labial base	smooth	smooth	ciliate	smooth	smooth	sometimes smooth	sometimes smooth	smooth*
Tip of l.p. to apex of papilla E	not reaching	almost reaching	not reaching	reaching	not reaching	not known	not known	not known
Chaeta m5 on Th. II	present	present	present	present	absent	present*	present	present*
Tip of tenent hair	capitate	pointed	capitate	capitate	capitate	capitate*	capitate	not known
Coxal macrochaetal formula	4 (3)/4+2, 3/4+2	4/4+1, 3/4+2	3/4+2, 3/4+2	3/4+1, 3/4+2	3/4+1, 3/4+2	3/4+1, 3/4+2	3/4(5)+2, 3/4+2	not known
Relative position of ms to sens on Abd. I	antero-external	antero-external	antero-external	antero-internal	antero-internal	not known	not known	not known
Relative length of normal sens to elongate sens on Abd. IV	about half	about half	not known	about half	almost equal	not known	not known	not known
Centro-posterior mac on Abd. IV	6 (A4, A5, A6, B4, B5, B6)	5 (A5, A6, B5, B6, Ae7)	7 (A4a, A4, A5, A6, B4, B5, B6)	6 (A4, A6, B4, B5, B6, Ae7)	3 (4) (A6, B6, Ae7, B5)	6–7 (A4, A5, A6, A4, B5, B6)	6 (A5, A6, Ae7, B4, B5, B6)	5–6 (A5, A6, B5, B6, Ae7) *

* based on Jordana’s description (2012).

#### ﻿ Homidia qianensis sp. nov.

Taxon classificationAnimaliaCollembolaEntomobryidae

D9675B10-5918-54E8-94BE-9E370F31C920

https://zoobank.org/F8D9C9A6-093F-4EF7-A90B-C947430F81DD

Figs 31–32 , 33–39 , 40–43 , 44 , 45, 46 , 47–53 , Table 2

##### Type material.

***Holotype*.** ♀ on slide, China, Guizhou Province, Liupanshui City, Pan County, Laochang Town, Shangkanzhe Village, 25°39'34″N, 104°48'28″E, 1677 m asl, sample number 1176, collected by H-D Tan, 6-II-2017, from bamboo litter, deposited in NTU. ***Paratype*.** ♀ on slide, same data as holotype.

##### Description.

Size. Body length up to 2.38 mm.

**Figures 31, 32.**
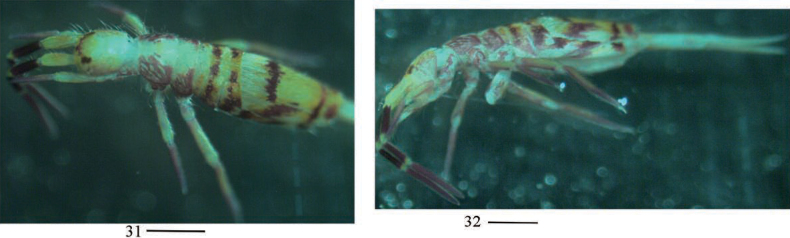
Habitus of *Homidiaqianensis* sp. nov. **31** dorsal view **32** lateral view. Scale bars: 500 μm.

***Coloration*.** Ground colour pale white or yellow. Ant. I pale yellow and Ant. II–IV blue. Eye patches dark blue and a transverse blue stripe between eye patches. Blue pigment present on dorsal head and Th. II–III laterally, legs, ventral tube and Abd. V. Abd. II–III with an irregular transverse blue stripe, respectively. Abd. IV with an irregular transverse blue stripe medially and a narrow blue stripe posteriorly (Figs 31, 32).

**Figures 33–39.**
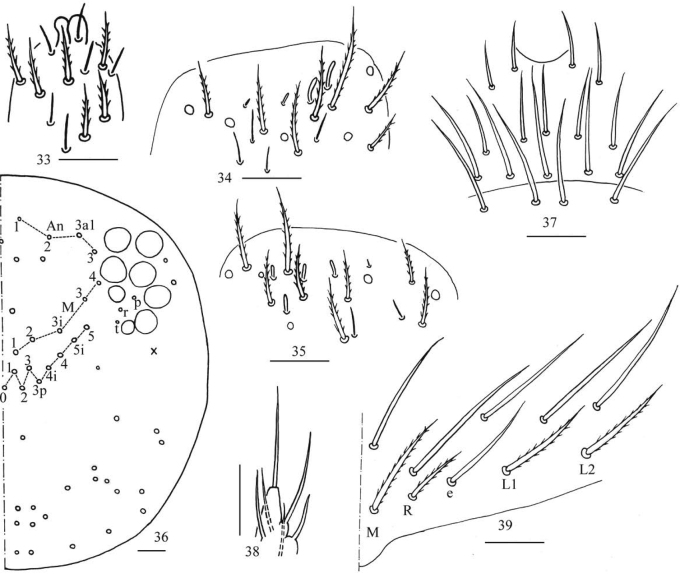
*Homidiaqianensis* sp. nov. **33** apex of Ant. IV **34**Ant. III organ **35** distal Ant. II **36** dorsal chaetotaxy of head **37** labrum **38** labial palp E **39** labial base. Scale bars: 20 μm.

**Figures 40–43.**
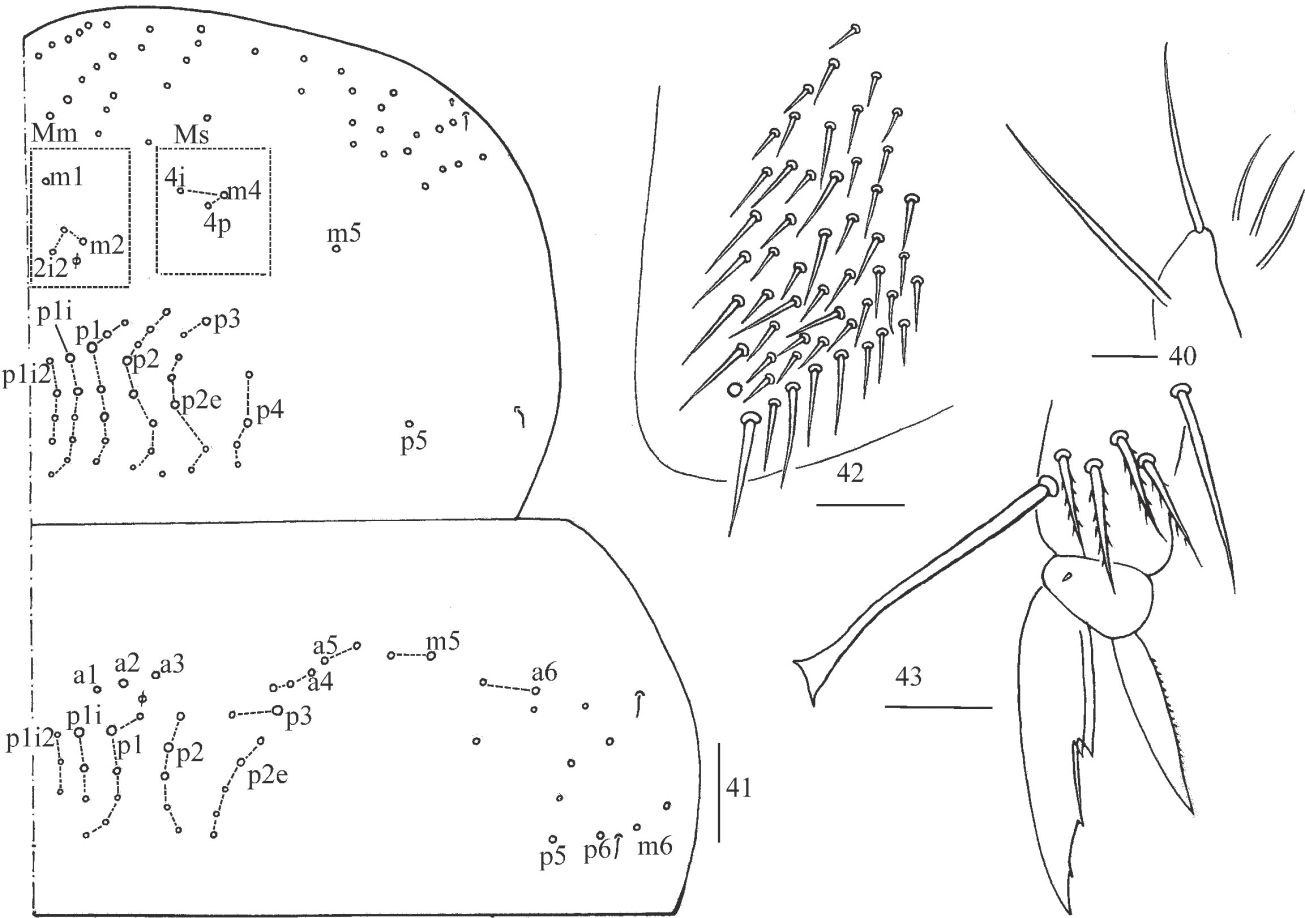
*Homidiaqianensis* sp. nov. **40** maxillary outer lobe **41** chaetotaxy of Th. II–III **42** trochanteral organ **43** hind foot complex. Scale bars: 20 μm (**40, 42, 43**); 50 μm (**41**).

***Head*.** Antenna 0.64–0. 69 times body length; antennal segment ratio I: II: III: IV = 1: 1.20–1.27: 1.05–1.09: 1.92–2.27. Apical bulb of Ant. IV bilobed (Fig. 33). Ant. III organ with two distal rod-like chaetae ventrally (Fig. 34). Ant. II with four distal rod-like chaetae ventrally (Fig. 35). Eyes 8 + 8, G and H smaller than others; interocular chaetae with p, r, and t mes. Dorsal cephalic chaetotaxy with four antennal (An1, An2, An3a1, An3), five median (M1, M2, M3i, M3, M4) and nine sutural (S0, S1, S2, S3, S3p, S4, S4i, S5i, S5) mac (Fig. 36). Labral chaetae as 4/5, 5, 4, all smooth; labral papillae not seen (Fig. 37). Lateral process (l.p.) of labial papilla E differentiated, as thick as normal chaeta, with tip almost reaching apex of papilla E (Fig. 38). Chaetal formula of labial base as MReL_1_L_2_, chaeta e smooth, others ciliate, R/M as 0.50–0.58 (Fig. 39). Basal chaeta of maxillary outer lobe thin, subequal to apical one; sublobal plate with three smooth chaeta-like processes (Fig. 40).

***Thorax*.**Th. II with four medio-medial (m1, m2, m2i, m2i2), three medio-sublateral (m4, m4i, m4p), 36–38 posterior mac, one ms and two sens (ms antero-internal to sens). Th. III with 47 mac and two sens (Fig. 41). Trochanteral organ with 53 smooth spiny chaetae (Fig. 42). Tenent hair capitate and almost subequal to inner edge of unguis in length. Unguis with four inner teeth and distal one very faint, basal pair located at 0.43–0.49 distance from base of inner edge of unguis, distal unpaired teeth at 0.65–0.70 and 0.87 distance from base, respectively; unguiculus lanceolate, outer edge slightly serrate (Fig. 43).

**Figure 44.**
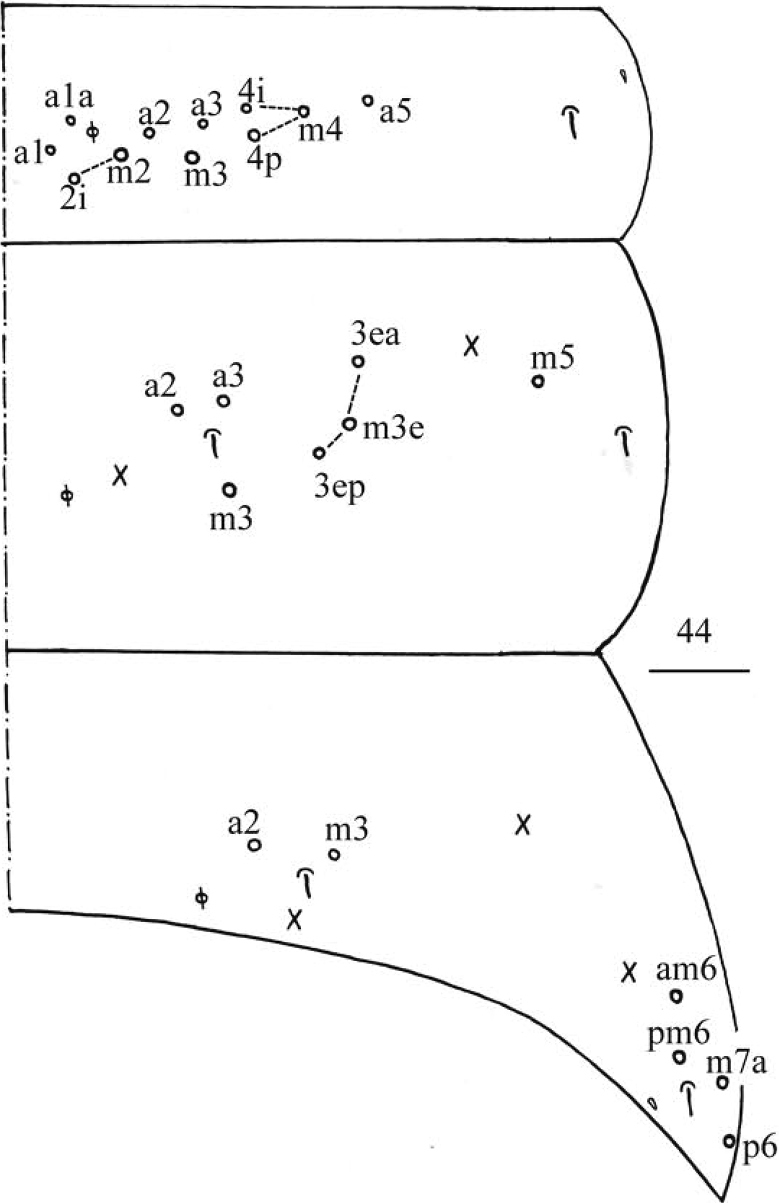
*Homidiaqianensis* sp. nov. Chaetotaxy of Abd. I–III. Scale bar: 50 μm.

**Figures 45, 46.**
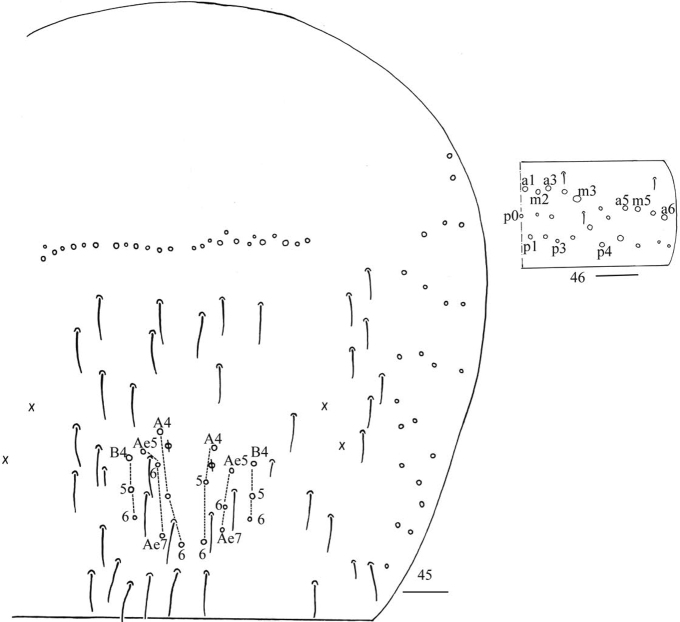
*Homidiaqianensis* sp. nov. **45** chaetotaxy of Abd. IV **46** chaetotaxy of Abd. V. Scale bars: 50 μm (**45**); 20 μm (**46)**.

**Figures 47–53.**
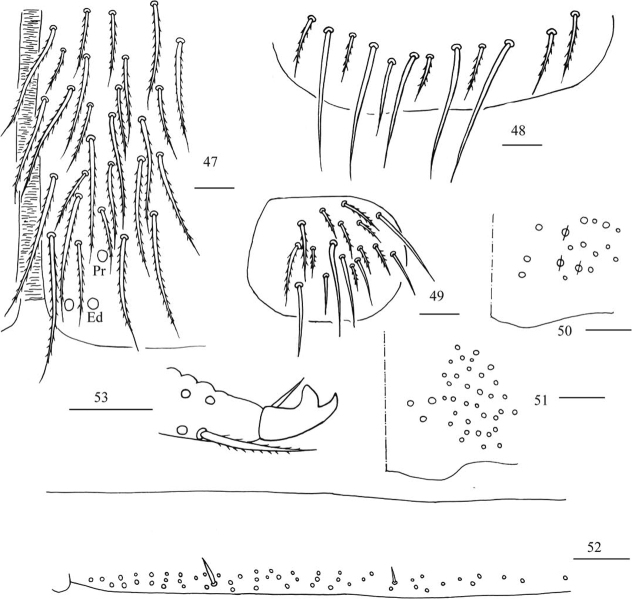
*Homidiaqianensis* sp. nov. **47** anterior face of ventral tube **48** posterior face of ventral tube apically **49** partial part of lateral flap of ventral tube **50** manubrial plaque **51** ventro-apical part of manubrium **52** proximal section of dens (circles also representing spines) **53** mucro. Scale bars: 20 μm.

***Abdomen*.** Range of Abd. IV length as 6.43–7.50 times as dorsal axial length of Abd. III. Abd. I with 11 (a1a, a1–3, a5, m2–4, m2i, m4i, m4p), ms antero-external to sens. Abd. II with six (a2, a3, m3, m3e, m3ea, m3ep) central, one (m5) lateral mac and two sens. Abd. III with two (a2, m3) central and four (am6, pm6, m7a, p6) lateral mac, one ms and two sens (Fig. 44). Abd. IV with two normal sens and about half length of elongate sens; anteriorly with 12–13 mac arranged in irregular transverse row, posteriorly with nine central mac (A4–6, B4–6, Ae5–7), laterally with 21–23 mac (Fig. 45). Abd. V with three sens, middle one posterior to m3 (Fig. 46). Anterior face of ventral tube with 30 ciliate chaetae, 3+3 of them as mac, line connecting proximal (Pr) and external-distal (Ed) mac oblique to median furrow (Fig. 47); posterior face with numerous ciliate chaetae and six smooth chaetae apically (Fig. 48); lateral flap not seen entirely (Fig. 49). Manubrial plate dorsally with 13–14 ciliate chaetae and three pseudopores (Fig. 50); ventrally with 36–37 ciliate chaetae on each side (Fig. 51). Dens with 54–57 smooth inner spines (Fig. 52). Mucro bidentate with subapical tooth larger than apical one; tip of basal spine reaching apex of subapical tooth; distal smooth section of dens almost equal to mucro in length (Fig. 53).

##### Etymology.

Named after its locality: Guizhou Province, which is abbreviated as Qian.

##### Ecology.

In the leaf litter of bamboo.

##### Remarks.

The new species is characterised by its colour pattern on the antennae and nine sutural mac on the head. Among 77 known species of the genus, 51 species have eight sutural mac and only nine species have nine sutural mac. There are some significant differences among them, such as colour pattern on the antennae and thorax, mac on Abd. III–IV and other characters, which are listed in Table 2.

**Table 2.** Comparison between *H.qianensis* sp. nov. and known species with nine sutural mac on the head.

Characters	*H.qianensis* sp. nov.	*H.flavonigra* Szeptycki, 1973	*H.grisea* Lee & Lee, 1981	*H.hexaseta* Pan, Shi & Zhang, 2011	*H.linhaiensis* Shi, Pan & Qi, 2009	*H.obliquistria* Ma & Pan, 2017	*H.pentachaeta* Li & Christiansen, 1997	*H.polyseta* Chen, 1998	*H.tiantaiensis* Chen & Lin, 1998	*H.ziguiensis* Jia, Chen & Christiansen, 2003
Colour pattern of antennae	Ant. I pale yellow, Ant. II–IV blue	Ant. I black proximally, Ant. II–IV yellow	Ant. I–IV gray proximally	antennae without blue pigment	Ant. III–IV with scattered blue pigment	brown pigment present on Ant. I–II distally and Ant. III–IV	blue pigment absent on Ant. I–II and present on Ant. III–IV	blue pigment absent on Ant. I–II and present on Ant. III–IV	blue pigment absent on Ant. I–II and present on Ant. III–IV	blue pigment present on antennae entirely except joints
Colour pattern on Th. II–III	Th. II–III with blue pigment laterally	Th. II–III almost black entirely	patches absent	patches absent	Th. II with blue pigment laterally and Th. III laterally and medially	Th. II–III with brown pigment laterally and medially	Th. III with a pair of patches medially	Th. II–III with three dark bands laterally and medially	Th. II with spots anteriorly and posteriorly and Th. III sublaterally	Th. II–III with blue pigment laterally and medially
Chaeta L_1_ on labial base	ciliate	smooth	smooth	smooth	smooth	ciliate	smooth	expanded	smooth	ciliate
Expanded chaetae on mentum	absent	absent	absent	absent	absent	present	absent	present	absent	present
Central and lateral mac on Abd. III	2, 4	3, unknown	2, 3	2, 4	2, 5	2, 4	3, 5	2, 4	2, 4	2, 4
Centro-anterior mac on Abd. IV	12–13	unknown	8–9	10–15	9–13	15–24	10–13	22–24	8–12	12–17
Centro-posterior mac on Abd. IV	9	13 or 15	4	9(10)	10–16	19(14)–32	12–16	25–30	23–27	9–16

#### ﻿ Entomobrya shaanxiensis sp. nov.

Taxon classificationAnimaliaCollembolaEntomobryidae

9EE44498-69F6-516A-9C1C-4631EA7E1114

https://zoobank.org/7085F164-CA63-4DEB-AB72-69AFFA791D80

Figs 54–55 , 56–60 , 61–63 , 64 , 65, 66 , 67–70 , Table 3

##### Type material.

***Holotype*.** ♀ on slide, China, Shaanxi Province, Xian City, Zhouzhi County, Cuifeng Town, Qingshan Park, 34°04'49″N, 108°01'58″E, 901 m asl, sample number 1109, collected by Y-T Ma, 17-VII-2012, from leaf litter, deposited in NTU. ***Paratypes*.** 4♀♀ on slides, same data as holotype.

##### Description.

Size. Body length up to 1.65 mm.

**Figures 54, 55.**
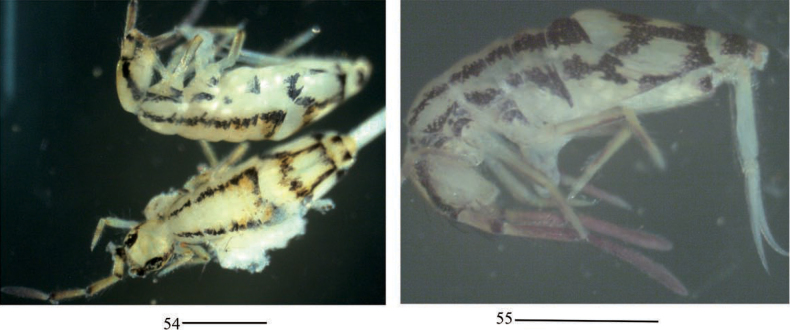
Habitus of *Entomobryashaanxiensis* sp. nov. Scale bars: 500 μm.

***Colouration*.** Ground colour pale yellow in ethanol. Ant. IV and distal part of Ant. I–III blue pigment. Eyepatches dark blue. An irregular blue stripe present from eyepatch to Th. III laterally and from Th. II to Abd. III sublaterally, respectively. Posterior part of Abd. III with a transverse irregular blue stripe. Abd. IV with two irregular transverse stripes, one located medially and other posteriorly. Abd. V with a pair of blue spots. Legs with scattered pigment (Figs 54, 55).

**Figures 56–60.**
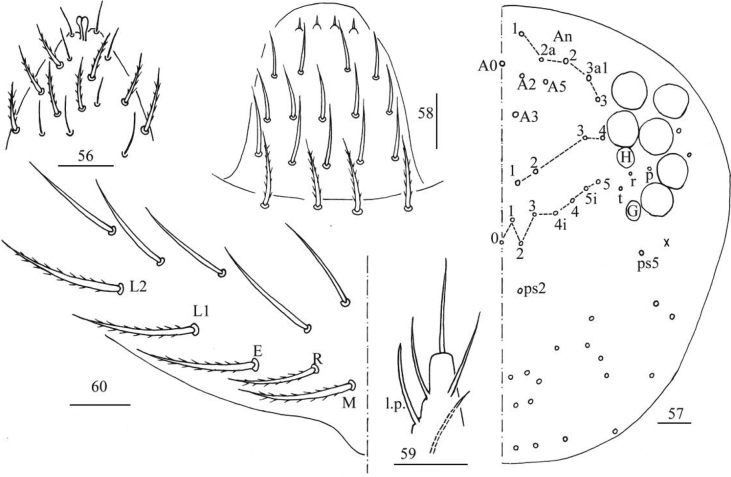
*Entomobryashaanxiensis* sp. nov. **56** apex of Ant. IV **57** dorsal chaetotaxy of head **58** labrum **59** labial palp E **60** labial base. Scale bars: 20 μm.

***Head*.** Antenna 0.47–0.55 times body length; antennal segment ratio I: II: III: IV = 1: 1.70–2.00: 1.50–1.88: 2.43–2.75. Apical bulb of Ant. IV bilobed (Fig. 56). Eyes 8 + 8, G and H smaller than others, interocular chaetae with p, r, and t mes. Dorsal cephalic chaetotaxy with five antennal (An1, An2a, An2, An3a1, An3), four median (M1, M2, M3, M4) and eight sutural (S0, S1, S2, S3, S4, S4i, S5i, S5) mac (Fig. 57). Labral chaetae 4/5, 5, 4, all slender; prelabral chaetae ciliate, other smooth, four labral papillae with one minute denticle each (Fig. 58). Lateral process (l.p.) of labial papilla E differentiated, as thick as normal chaeta, with tip exceeding apex of papilla E (Fig. 59). Chaetal formula of labial base as MREL_1_L_2_, M rarely duplicate, all ciliate; R 0.56–0.71 times length of M (Fig. 60).

**Figures 61–63.**
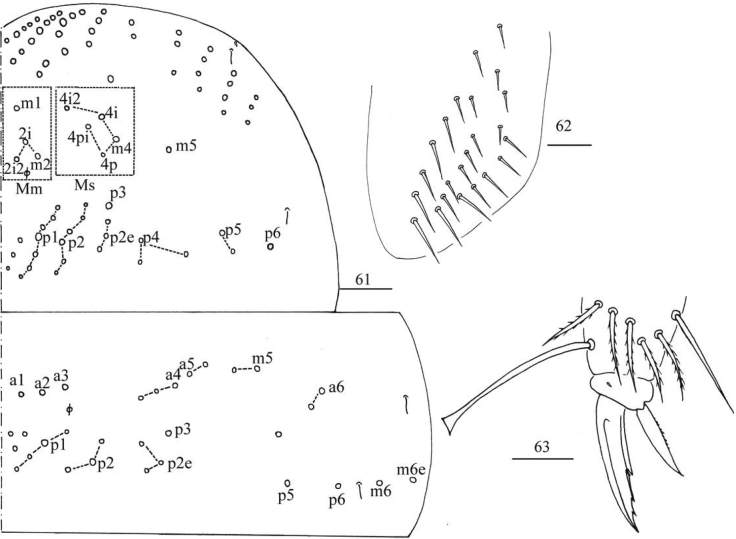
*Entomobryashaanxiensis* sp. nov. **61** chaetotaxy of Th. II–III **62** trochanteral organ **63** hind foot complex. Scale bar: 50 μm (**61**); Scale bars: 20 μm (**62, 63**).

***Thorax*.**Th. II usually with four medio-medial (m1, m2, m2i, m2i2 rarely absent), four medio-sublateral (m4, m4i, m4p, m4pi, m4i2 rarely present), 23–27 (p6 sometimes absent) posterior mac, one ms and two sens (ms antero-external to sens). Th. III with 29–33 mac and two sens (Fig. 61). Trochanteral organ with 21–27 smooth spiny chaetae (Fig. 62). Tenent hair capitate and longer than inner edge of unguis in length. Unguis with four inner teeth. Unguiculus acuminate and outer edge slightly serrate (Fig. 63).

**Figure 64.**
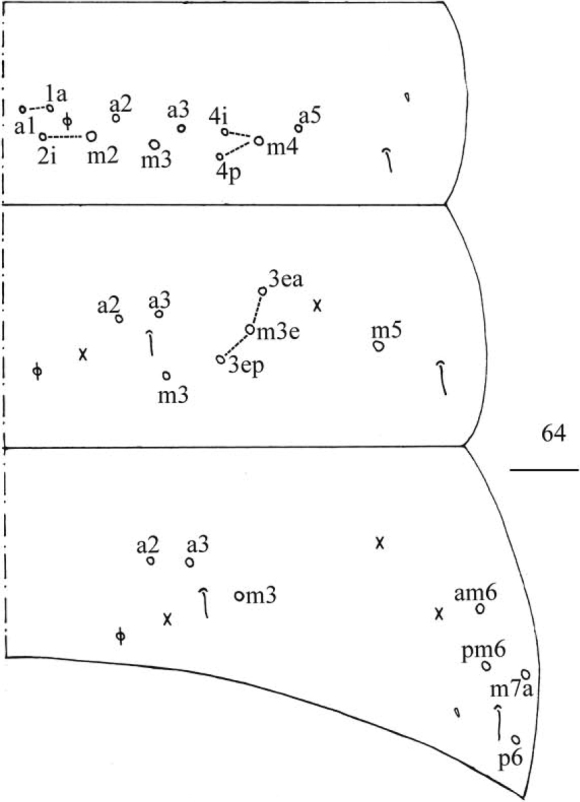
*Entomobryashaanxiensis* sp. nov. Chaetotaxy of Abd. I–III. Scale bar: 50 μm.

**Figures 65, 66.**
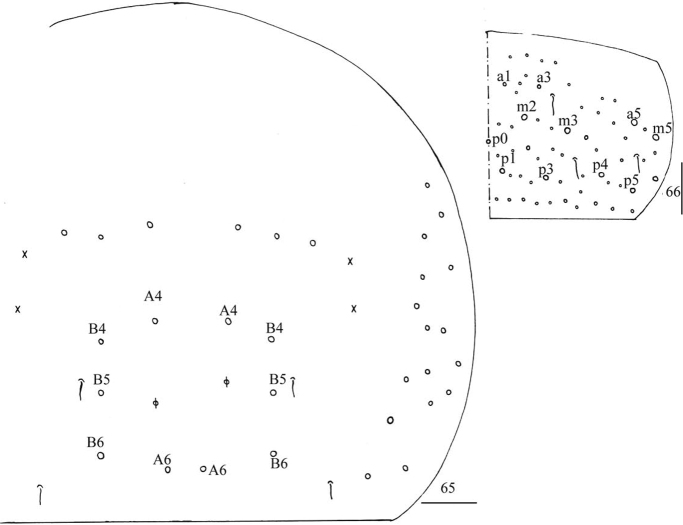
*Entomobryashaanxiensis* sp. nov. **65** chaetotaxy of Abd. IV **66** chaetotaxy of Abd. V. Scale bars: 50 μm (**65**); 20 μm (**66**).

**Figures 67–70.**
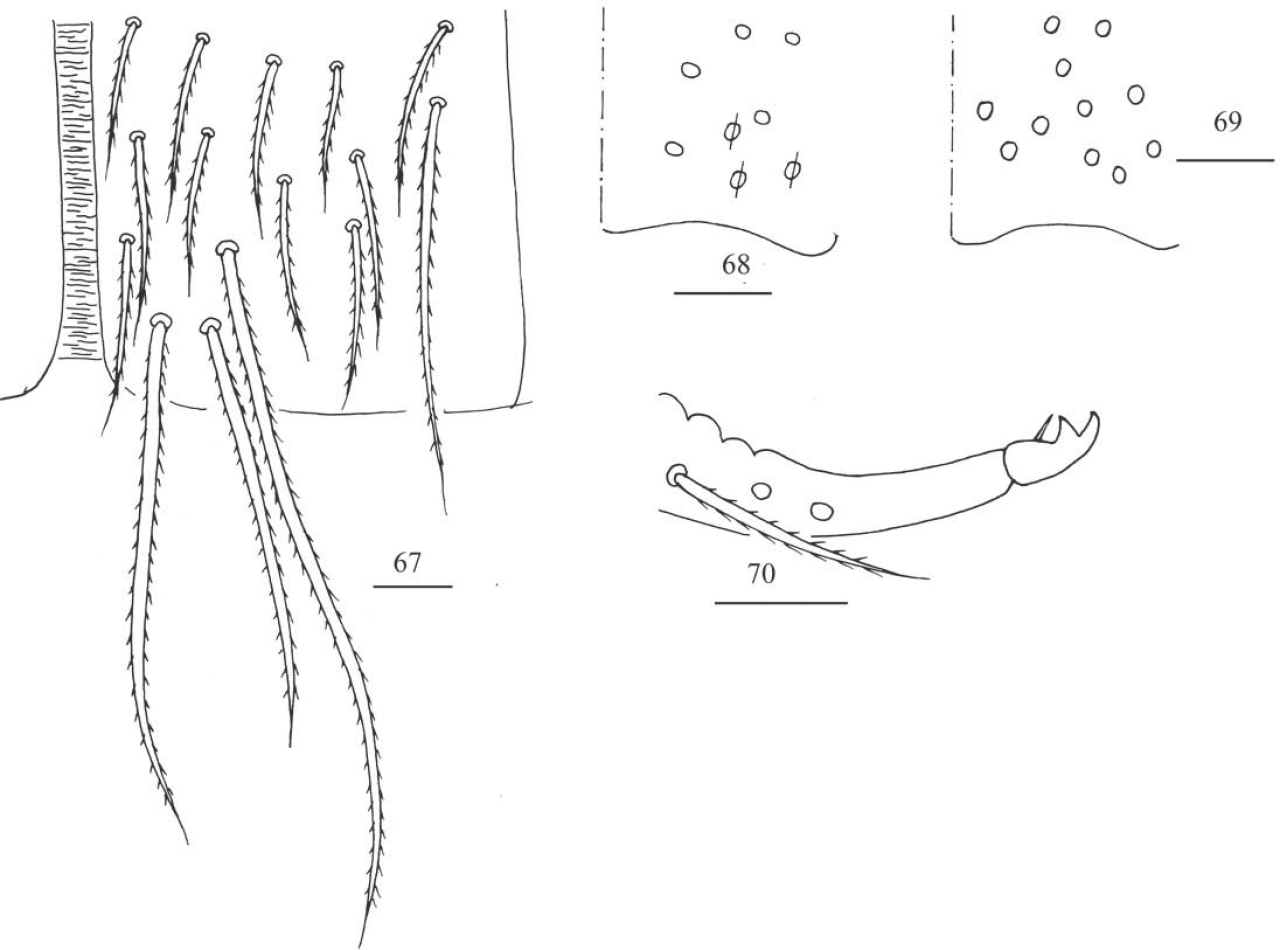
*Entomobryashaanxiensis* sp. nov. **67** anterior face of ventral tube **68** manubrial plaque **69** ventro-apical part of manubrium **70** mucro. Scale bars: 20 μm.

***Abdomen*.** Range of Abd. IV length as 4.03–5.50 times dorsal axial length of Abd. III. Abd. I with 10–11 (a1, a1a, a2–3, m2–4, m2i, m4i, m4p, a5, a1a sometimes absent) mac, ms antero-external to sens. Abd. II with six (a2, a3, m3, m3e, m3ea, m3ep) central, one (m5) lateral mac and two sens. Abd. III with two (a2, a3) central, and four (am6, pm6, m7a, p6) lateral mac, one ms and two sens (Fig. 64). Abd. IV with two normal length sens; centrally with eight mac, laterally 13–16 mac (Fig. 65). Abd. V with three sens, middle one posterior to m3 (Fig. 66). Anterior face of ventral tube with some ciliate chaetae, 3+3 of them as mac, line connecting proximal (Pr) and external-distal (Ed) mac oblique to median furrow (Fig. 67); posterior face and lateral flap not clearly seen. Manubrial plaque dorsally with about five ciliate chaetae and three pseudopores (Fig. 68); ventrally with 11–13 ciliate chaetae (Fig. 69). Distal smooth part of dens about 2.33 times as long as mucro. Mucro bidentate with subapical tooth subequal to apical one; tip of basal spine reaching apex of subapical tooth (Fig. 70).

##### Etymology.

Named after its locality: Shaanxi Province.

##### Ecology.

In the leaf litter.

##### Remarks.

The new species is characterised by its colour pattern, one minute denticle on each labral papilla, and the tip of the lateral process (l.p.) of the labial papilla E exceeding the apex of the papilla E. It is very similar to *E.aino* (Matsumara & Ishida, 1931) in colour pattern, macrochaetotaxy of Abd. III–IV and other characters, but there are some differences between them, such as chaetotaxy on Abd. I, II and IV (Table 3).

**Table 3.** Comparison between *E.shaanxiensis* sp. nov. and *E.aino*.

Characters	*E.shaanxiensis* sp. nov.	*E.aino* (Matsumara & Ishida, 1931)
Longitudinal stripe on midline from Th. III–Abd. II	absent	present^#^
Pigment on Th. II–III laterally	present	absent^#^
Denticle on labral papilla	present	absent^#^
R chaeta on labial base	not duplicate	duplicate^#^
Medio-sublateral mac on Th. II	4 (rarely 5)	2–3*
Mac on Abd. I	10–11	13^#^
Mac m3ei on Abd. II	absent	present^#^
Mac on Abd. IV centrally	8	5*

^#^ based on Lee and Park’s description (1992); * based on Jordana’s description (2012).

#### ﻿ Akabosia matsudoensis

Taxon classificationAnimaliaCollembolaEntomobryidae

Kinoshita, 1919

84A08E49-1CD5-53D8-8807-E164F782A72B

Figs 71–72 , 73–78 , 79–81 , 82–87 , 88–89

Akabosia matsudoensis Kinoshita, 1919: 16–20.

##### Examined specimens.

5♀♀ on slides, China, Jiangsu Province, Nantong City, Intersection of Tongning Highway and Pingning Road, 32°04'21″N, 120°50'19″E, sample number 1249, collected by Y-T Ma, 1-IX-2022, in the leaf litter of *Salixbabylonica*.

##### Description of specimens from China.

Size. Body length up to 1.81 mm.

**Figures 71, 72.**
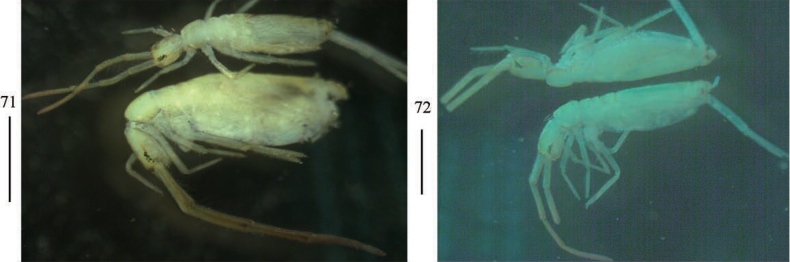
Habitus of *Akabosiamatsudoensis*. Scale bars: 500 μm.

***Coloration*.** Ground colour pale white to pale yellow. Eye patches dark blue. A little brown pigment present on antennae and Abd. V (Figs 71, 72).

**Figures 73–78.**
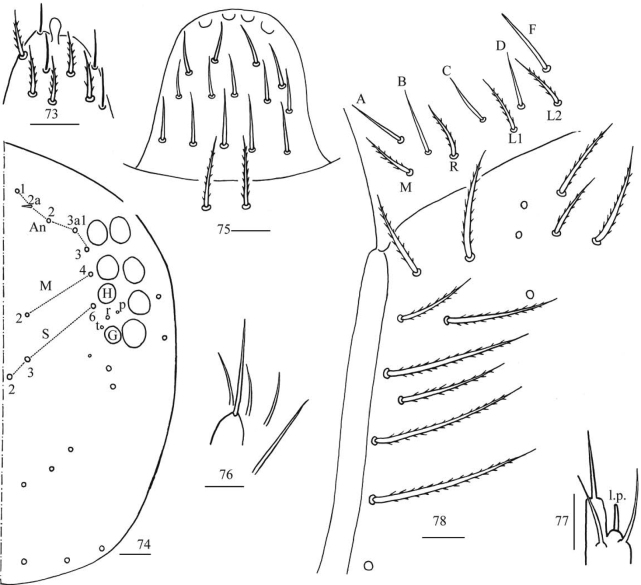
*Akabosiamatsudoensis***73** apex of Ant. IV **74** dorsal chaetotaxy of head **75** labrum **76** maxillary outer lobe **77** labial palp E **78** labial and posterior labial chaetae. Scale bars: 20 μm.

***Head*.** Antenna 0.70–1.03 times body length; antennal segment ratio I: II: III: IV = 1: 1.10–1.34: 1.10–1.40: 1.60–1.83. Apical bulb of Ant. IV simple (Fig. 73). Eyes 8 + 8, G and H smaller than others; interocular chaetae with p, r, and t mes. Dorsal cephalic chaetotaxy with five antennal (An1, An2a, An2, An3a1, An3), two median (M2, M4), and three sutural (S2, S3, S6) mac (Fig. 74). Labral chaetae as 2/5, 5, 4, prelabral chaetae ciliate, other smooth; labral margin with four papillae (Fig. 75). Basal chaeta of maxillary outer lobe thin, subequal to apical one; sublobal plate with three smooth chaeta-like processes (Fig. 76). Lateral process (l.p.) of labial papilla E differentiated, as thick as normal chaeta, with tip not reaching apex of papilla E (Fig. 77). Labium with ABCDF chaetae, all smooth, chaeta F apically blunt; chaetae of labial base as MRL_1_L_2_, all ciliate (Fig. 78).

**Figures 79–81.**
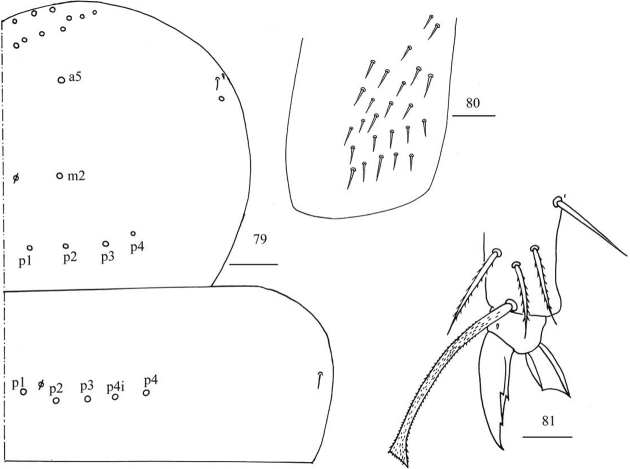
*Akabosiamatsudoensis***79** chaetotaxy of Th. II–III **80** trochanteral organ **81** hind foot complex. Scale bars: 50 μm (**79**); 20 μm (**80, 81**).

***Thorax*.**Th. II with one medio-medial (m2), three (p1–3, p4 rarely present) posterior mac, one ms and one sens (ms antero-enternal to sens). Th. III with five (p1–4, p4i) mac and one sens (Fig. 79). Trochanteral organ with 25–31 smooth spiny chaetae (Fig. 80). Tenent hair ciliate and longer than inner edge of unguis, with tip capitate. Unguis with three inner teeth, basal pair located at 0.41–0.50 distance from base of inner edge of unguis, distal one small and at 0.75–0.76 distance from base; inner lamellae of unguiculus truncate, external lamellae acuminate (Fig. 81).

**Figures 82–87.**
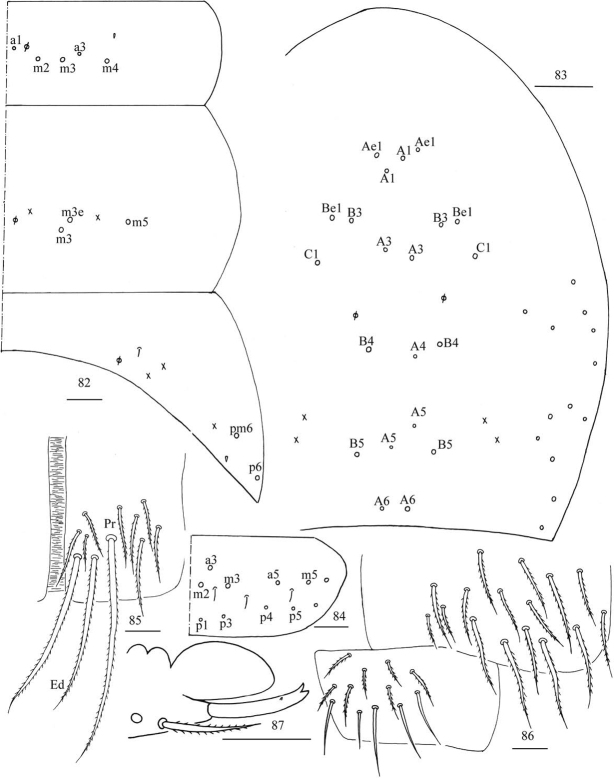
*Akabosiamatsudoensis***82** chaetotaxy of Abd. I–III **83** chaetotaxy of Abd. IV **84** chaetotaxy of Abd. V **85** anterior face of ventral tube **86** posterior face and lateral flap of ventral tube **87** mucro. Scale bars: 50 μm (**82, 83**); 20 μm (**84–87**).

**Figures 88, 89.**
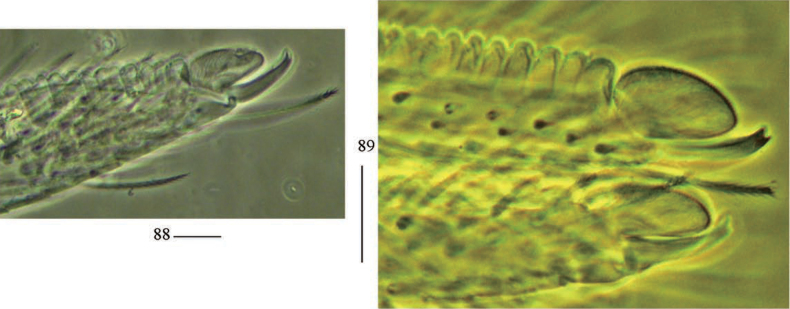
Mucro of *Akabosiamatsudoensis*. Scale bars: 20 μm.

***Abdomen*.** Range of Abd. IV length as 9.21–10.45 times dorsal axial length of Abd. III. Chaetotaxy Abd. I–III as in Fig. 82. Abd. I with five (a1, a3, m2–4, a1 rarely absent, a3 sometimes absent), one ms. Abd. II with two (m3, m3e) central, one (m5) lateral mac. Abd. III with two (pm6, p6) lateral mac, one ms and one sens. Lateral part of Abd. IV with 10–12 mac. Middle part of Abd. IV with two (A1, Ae1) mac anteriorly; four (A3, B3, Be1, C1) mac centrally; four or five (B4, B5, A6, A4 and A5 sometimes absent) posteriorly (Fig. 83). Abd. V with three sens, middle one posterior to m3 (Fig. 84). Anterior face of ventral tube with 11–15 ciliate chaetae, 3+3 of them as mac, line connecting proximal (Pr) and external-distal (Ed) mac oblique to median furrow (Fig. 85); posterior face with many ciliate chaetae; lateral flap with five or eight smooth and seven or 24 ciliate chaetae (Fig. 86). Mucro rectangle and with two teeth apically and one small tooth medio-distally (Figs 87–89).

##### Remarks.

The genus *Akabosia* was established by Kinoshita in 1919 and it can be separated from its similar genus *Salina* by the crenulated dens (dens is not crenulated in *Salina*). Only one species, *A.matsudoensis* Kinoshita, 1919, has been reported in the genus and its localities include Japan (Kinoshita 1919; Yosii 1954, 1965), Korea (Mitra 1977) and China (Zhang et al. 2015). The characters of our specimens agree well with their description in colour pattern, chaetotaxy of dorsal body, labrum and mucro, and we describe some additional characters including maxillary outer lobe, labial papilla E, chaetotaxy of Abd. V for the first time. The medio-distal tooth on the mucro was overlooked previously as it is very small and located on the lateral side.

## Supplementary Material

XML Treatment for Homidia pseudozhangi

XML Treatment for Homidia qianensis

XML Treatment for Entomobrya shaanxiensis

XML Treatment for Akabosia matsudoensis

## ﻿Additional information

### Author ORCIDs

Mei-Dong Jing https://orcid.org/0000-0002-8498-9498

Yi-Tong Ma https://orcid.org/0000-0002-8660-0503

### Data availability

All of the data that support the findings of this study are available in the main text or Supplementary Information.
